# A new EEG neurofeedback training approach in sports: the effects function-specific instruction of Mu rhythm and visuomotor skill performance

**DOI:** 10.3389/fpsyg.2023.1273186

**Published:** 2023-12-22

**Authors:** Kuo-Pin Wang, Ming-Yang Cheng, Hatem Elbanna, Thomas Schack

**Affiliations:** ^1^Center for Cognitive Interaction Technology (CITEC), Bielefeld University, Bielefeld, Germany; ^2^Neurocognition and Action - Biomechanics Research Group, Faculty of Psychology and Sports Science, Bielefeld University, Bielefeld, Germany; ^3^School of Psychology, Beijing Sport University, Beijing, China; ^4^Department of Sports Psychology, Faculty of Physical Education, Mansoura University, Mansoura, Egypt

**Keywords:** complex visuomotor skills, simple visuomotor skills, Mu rhythm, alpha rhythm, mental training, golf putting

## Abstract

**Introduction:**

Achieving optimal visuomotor performance in precision sports relies on maintaining an optimal psychological state during motor preparation. To uncover the optimal psychological state, extensive EEG studies have established a link between the Mu rhythm (8–13 Hz at Cz) and cognitive resource allocation during visuomotor tasks (i.e., golf or shooting). In addition, the new approach in EEG neurofeedback training (NFT), called the function-specific instruction (FSI) approach, for sports involves providing function-directed verbal instructions to assist individuals to control specific EEG parameters and align them with targeted brain activity features. While this approach was initially hypothesized to aid individuals in attaining a particular mental state during NFT, the impact of EEG-NFT involving Mu rhythm on visuomotor performance, especially when contrasting the traditional instruction (TI) approach with the FSI approach, underscores the necessity for additional exploration. Hence, the objective of this study is to investigate the impact of the FSI approach on modulating Mu rhythm through EEG-NFT in the context of visuomotor performance.

**Methods:**

Thirty novice participants were recruited and divided into three groups: function-specific instruction (FSI, four females, six males; mean age = 27.00 ± 7.13), traditional instruction (TI, five females, five males; mean age = 27.00 ± 3.88), and sham control (SC, five females, five males; mean age = 27.80 ± 5.34). These groups engaged in a single-session EEG-NFT and performed golf putting tasks both before and after the EEG-NFT.

**Results:**

The results showed that within the FSI group, single-session NFT with augmented Mu power led to a significant decrease in putting performance (*p* = 0.013). Furthermore, we noted a marginal significance indicating a slight increase in Mu power and a reduction in the subjective sensation of action control following EEG-NFT (*p* = 0.119). While there was a positive correlation between Mu power and mean radial error in golf putting performance (*p* = 0.043), it is important to interpret this relationship cautiously in the context of reduced accuracy in golf putting.

**Discussion:**

The findings emphasize the necessity for extended investigation to attain a more profound comprehension of the nuanced significance of Mu power in visuomotor performance. The study highlights the potential effectiveness of the FSI approach in EEG-NFT and in enhancing visuomotor performance, but it also emphasizes the potential impact of skill level and attentional control, particularly in complex visuomotor tasks.

## Introduction

Optimizing visuomotor performance requires individuals to achieve and maintain an optimal psychological state during motor preparation (Krane and Williams, 2006). In visuomotor tasks, such as golf putting and shooting, motor programming is a crucial psychological construct that involves organizing and controlling the various degrees of freedom in movement to execute a skill (Schmidt et al., 2018). Successful motor programming leads to superior visuomotor performance by allowing individuals to execute appropriate motor control, such as movement force, direction, and stability (Cooke et al., 2014; Wang et al., 2019, 2022; Chen et al., 2022a). The regulation of motor programming processes during skill execution significantly impacts visuomotor performance, making it essential to identify innovative approaches to refine these processes (Cooke et al., 2015; Bertollo et al., 2016; Chang and Hung, 2020).

Prior research utilizing electroencephalograms (EEG) has established a link between motor programming and motor performance. Specifically, the Mu rhythm, identified within the 8–13 Hz frequency range in the central brain region (Cooke et al., 2014, 2015), is significant in the context of motor programming. This rhythm is indicative of the allocation of cognitive resources during both the observation and execution phases of goal-directed actions (Pineda, 2005; Cannon et al., 2014; Cooke et al., 2015). It serves as a neural marker for understanding the association between motor programming processes and motor preparation for motor actions, emphasizing its role in both the planning and implementation stages of movement. Mu rhythm activity has been found to influence visuomotor performance during golf putting (Babiloni et al., 2008; Cooke et al., 2014; Wang et al., 2019, 2020, 2022) and shooting (Haufler et al., 2000; Del Percio et al., 2009; Bertollo et al., 2016) in the field of sport psychophysiology. However, the physiological function of the Mu rhythm during visuomotor actions remains a topic of ongoing scientific debate. Specifically, Kerick et al. (2004) have found that increased Mu power in the central region leads to improved shooting performance, indicating deactivation of the central area, which may be associated with adaptive sensorimotor integration and less cognitive effort due to automaticity (Kober et al., 2015; Cheng et al., 2017). That is, increased Mu power may reflect less allocation of irrelevant cognitive resources to response motor programming for psychomotor efficiency and to exemplify a refinement of neural processes, consistent with the stage of automaticity (Cheng et al., 2015b; di Fronso et al., 2016; Hatfield, 2018). However, these findings contrast with other studies that have reported an opposite relationship between Mu rhythm and motor performance. For instance, recent research has suggested that decreased Mu power is associated with superior performance in a golf task (Cooke et al., 2014; Wang et al., 2019, 2020), suggesting higher cognitive resources to response motor programming, leading to adaptive motor control and increased action control levels during golf tasks (Wang et al., 2023). Given these conflicting results in the visuomotor tasks (i.e., shooting and golf), further investigation is needed to clarify the role of Mu rhythm in visuomotor tasks, which may offer a more precise understanding of the physiological function of motor programming in visuomotor performance.

Recent studies have been using EEG neurofeedback training (NFT) to clarify the role of EEG activity (Kao et al., 2014; Cheng et al., 2015a; Ring et al., 2015; Chen et al., 2022b; Wang et al., 2023). EEG-NFT is a tool to indirectly affect brain function and link brain with psychological states for sports performance (Cooke et al., 2018; Cheng and Hung, 2020a; Onagawa et al., 2023). For example, a decrease in frontal midline theta (FMT; 4–7 Hz) power that is linked to sustained attention (Kao et al., 2014) can improve putting performance in skilled golfers (Chen et al., 2022b). In addition, increased sensorimotor rhythm (SMR; 12–15 Hz) power that is associated with attentional processing can improve putting performance in skilled golfers (Cheng et al., 2015a; Afrash et al., 2023) and novice golfers (Pourbehbahani et al., 2023). In addition to FMT and SMR, Mu rhythm can also modulate visuomotor performance. Wang et al. (2023) conducted a first randomized controlled trial study to explore the influence of Mu rhythm modulation on visuomotor performance. They recruited 30 novice golfers divided into three groups: increased Mu rhythm, decreased Mu rhythm, and sham control and performed a golf putting task. The findings indicated that reduction in Mu power in the decreased Mu rhythm group resulted in increased subjective sensation of action control level and improved performance. However, this finding is inconsistent with Kerick et al. (2004), who reported that an increase in Mu power can cause performance improvement in shooting task. Wang et al. (2023) highlighted that the complexity of motor skills may influence the Mu power and visuomotor performance. This observation aligns with Berka et al. (2010) distinction between complex visuomotor skills and simple visuomotor skills, such as golf and shooting. Compared to simple skills, complex skills require more cognitive resources and involve intricate neural processes to achieve superior performance (Cooke et al., 2015; Afrash et al., 2023). As a result, a decrease in Mu power may have a positive impact on performance in complex visuomotor task (Afrash et al., 2023; Wang et al., 2023). Nevertheless, Wang et al. (2023) suggested that the association between Mu rhythm and performance is still ambiguous in the visuomotor task because no significant changes in Mu power were observed in the increased Mu rhythm group. The failed Mu power manipulation in the increased Mu rhythm group may be because of a lack of specific verbal instructions for individuals to learn how to increase Mu power during EEG-NFT (Chen et al., 2022b). Therefore, it is important to use specific instructions for EEG-NFT execution to better understand the physiological function of Mu rhythm in visuomotor performance.

To address the need for specific instructions in EEG-NFT, the utilization of the function-specific instruction (FSI) approach in EEG-NFT for sports has been explored in a study that conducted by Chen et al. (2022b). FSI approach can be used to address the inconsistent findings in previous studies (Kerick et al., 2004; Wang et al., 2023). This approach provides function-directed verbal instructions for participants to control specific EEG parameters, aiming to align the instructions with targeted brain activity features during EEG-NFT (deCharms et al., 2005; Chen et al., 2022b). Specifically, the FSI approach considers the meaning of the brainwave function in the target region and the EEG power magnitude to attain a specific mental state. A previous study has provided evidence of the positive effects of the FSI approach in EEG-NFT for sport performance improvement (Chen et al., 2022b; Wu et al., 2023). For instance, Chen et al. (2022b) recruited 36 skilled golfers, which were divided into three groups: FSI, traditional instruction (TI), and sham control (SC), and measured their putting performance before and after performing EEG-NFT. The FSI group demonstrated a significant improvement in putting performance and decrease in FMT power. These findings suggest that the FSI approach is more effective than TI approach in manipulating EEG activity, enhancing sustained attention and putting performance in skilled golfers (Chen et al., 2022b). However, despite these findings, little is known about the effects of Mu activity with the FSI approach in EEG-NFT. Mu activity has been demonstrated to function as an indicator of cognitive resource allocation through conscious effort during preparation, consequently influencing motor performance (Cooke et al., 2015; Wang et al., 2023). By incorporating the FSI approach, Mu NFT can potentially alter visuomotor performance. Therefore, adopting the FSI approach in Mu NFT can provide insights into targeted interventions for enhancing visuomotor performance in sports and other motor skills.

The objective of the current study is to examine if implementing the FSI approach can improve the efficacy of Mu NFT and its effect on visuomotor performance. To do this, we replicate the study that conducted by Wang et al. (2023) who recruited novice golfers and adopted single-session EEG-NFT to examine Mu activity impact on complex visuomotor performance, especially golf putting performance (Kao et al., 2014; Chen et al., 2022a). Although multi-session interventions in EEG-NFT may increase the effectiveness of learning outcomes, such as three (Arns et al., 2008), six (Afrash et al., 2023), and eight (Cheng et al., 2015a) sessions, a single-session intervention in EEG-NFT with an FSI approach has been found to be sufficient for individuals to learn to control neural activity in the brain (Kao et al., 2014; Chen et al., 2022b; Wu et al., 2023). In addition, a single-session intervention in EEG-NFT provides a possibility for practical application in sports (Hung and Cheng, 2018; Cheng and Hung, 2020b; Wang et al., 2023). Accordingly, the study aims to complement the findings of Wang et al. (2023) by manipulating Mu rhythm during a golf putting task in a single-session EEG-NFT and examining whether increased Mu power (i.e., a decrease in cortical activity), which is likely associated with reduced cognitive effort (Kerick et al., 2004), could result in improved or decreased performance in a golf putting task. To test our hypothesis, we established three groups following the previous protocol (Chen et al., 2022b): an FSI group, a TI group, and a SC group. We hypothesize that the FSI group could exhibit a more significant alteration in performance in a golf putting task (i.e., a complex visuomotor task) than the TI and SC groups after a single-session Mu NFT. Additionally, we hypothesize that the FSI group will exhibit significantly increased Mu power after a single-session Mu NFT than the other groups.

## Materials and methods

### Participants

A power analysis was conducted to determine the minimum detectable effect for a repeated measure, within-between interaction, multivariate analysis of variance (MANOVA) sample size calculation using G^*^Power, in accordance with the guidelines established by Faul et al. (2007). The study utilized values of α = 0.05, power = 0.80, effect size = 0.70 (corresponding to $\eta_{p}^{2}$ = 0.33), with three groups (TI, FSI, and SC) and eight measurements (pre-post measurements × electrode sites) in a priori type of power analysis, following the research design employed by Wang et al. (2023). We chose Wilks U in the approximation (F-transformation; Rao, 1951) and O'Brien and Shieh (1999), recommended. The minimum sample size required was determined to be *N* = 26. To mitigate potential biases arising from power analysis, which has been highlighted in the neuroscience field (Albers and Lakens, 2018; Algermissen and Mehler, 2018), a total of 30 novices were recruited in three groups. All participants were assigned to the TI (five females, five males; mean age = 27.00 ± 3.88), FSI four females, six males; mean age = 27.00 ± 7.13), and S (five females, five males; mean age = 27.80 ± 5.34), respectively. All eligible participants were screened based on the following criteria: (1) no history of psychiatric or neurological disease; (2) right-handed; (3) not taking medication affecting the central nervous system or brain; (4) normal or corrected-to-normal vision; and (5) normal visual attention that was assessed by using Trail Making Test A (Lezak et al., 2004). Informed consent was obtained from all participants before the study commenced. This study was approved by the institutional review board of Bielefeld University, and all procedures and methods were conducted in accordance with the relevant ethical guidelines and regulations.

### Measures

#### Golf putting task

**Figure 1:**
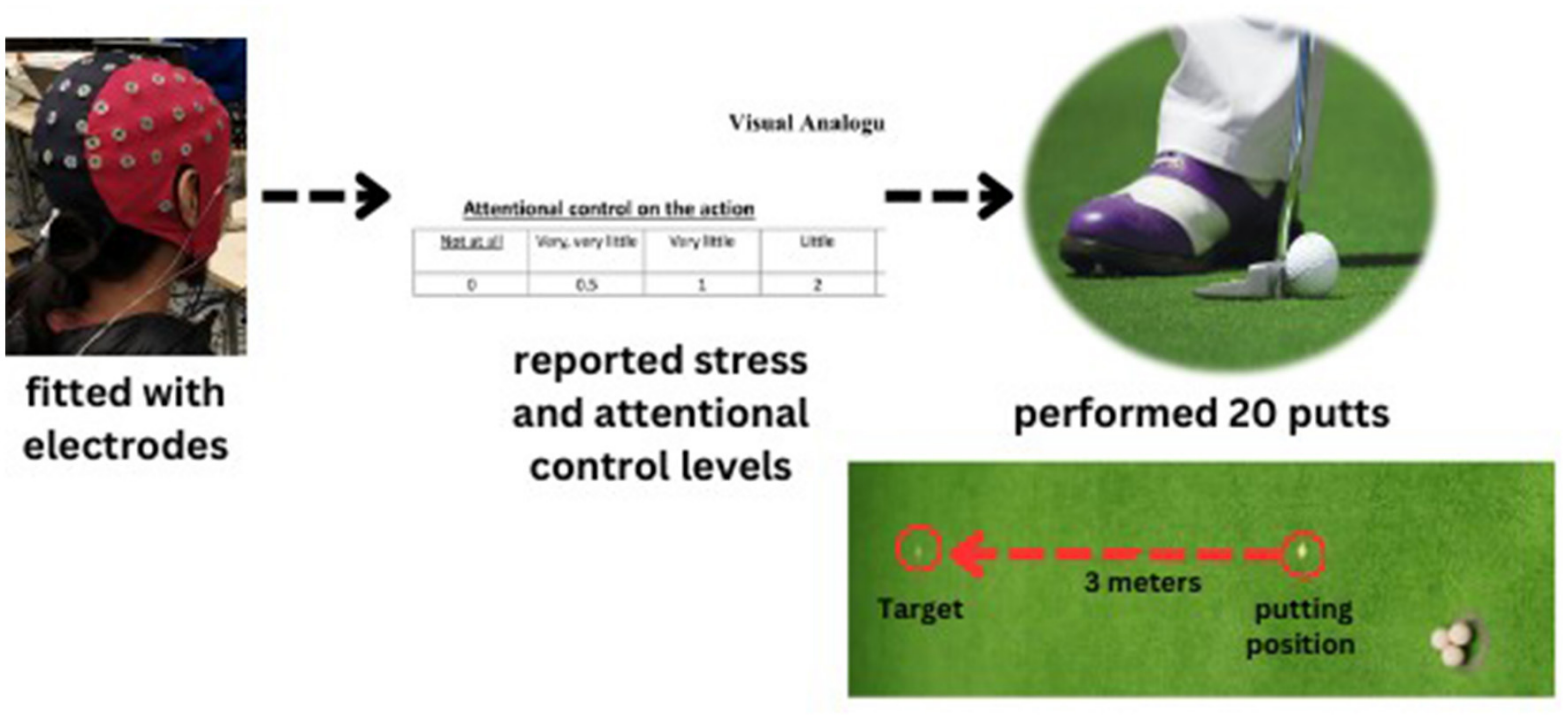
The golf putting task and procedure.

The participants employed a standard putter suitable for regular-sized golf balls (diameter = 4.27 cm) to execute putts aimed toward a target positioned 3 meters away from them on an artificial putting green (4 × 9 m). Both before and after the EEG-NFT intervention, the participants performed 20 putts (i.e., pretest-posttest). During the putting task, the definition of the motor preparation period was that specified by Wang et al. (2020), who defined it as the period between placing the putter behind the ball and initiating the backswing. For each trial, backswing movement was detected by an infrared sensor as an event marker (Figure 1).

### Subjective stress level

To address the potential influence of stress on the experimental outcomes (confounding effects), a subjective evaluation of stress levels was conducted using an 11-point Likert scale ranging from 0 (representing no stress) to 11 (indicating the highest level of stress), as previously described by Wang et al. (2023). This assessment was carried out during the golf putting task both before and after EEG-NFT.

#### Subjective psychological state (attentional control level)

Research suggests that the 8–13 Hz frequency range at the central cortex (Cz) is associated with both motor programming in neuromotor process and attentional control of actions (Wang et al., 2023). To assess participants' attentional control during the golf putting task, they were asked to rate their sensation of action control level on an 11-point Likert scale ranging from 0 (not at all) to 11 (maximum possible; Wang et al., 2022) before and after EEG-NFT intervention. Specifically, we asked participants to report the number on the 11-point Likert scale before proceeding to perform 20 putts. Additionally, they reported the number when standing on the putting area in both the pre and posttests separately.

### Instrumentation

#### Vicon motion systems

A motion capture system (Vicon Motion Systems, Oxford, UK) was utilized to record putting performance. Specifically, the ball's movement was monitored using six T10 charge-coupled device cameras with a spatial resolution of ~0.25 mm and a temporal resolution of 200 Hz, recording its rolling and stopping.

#### EEG

In accordance with the international 10-10 system, 64 electrode sites were utilized to record data. Electrodes were placed on the left and right ear mastoids (M1, M2) to serve as the electrical reference and at the anterior frontal zone position (AFz; Jurcak et al., 2007) to serve as the ground electrode. In addition, bipolar configurations were placed superior and inferior to the left eye, and on the left and right orbital canthi to record vertical and horizontal electrooculograms (HEOL, HEOR, VEOU, and VEOL). The eego system (ANT Neuro, Germany) was used with a bandpass filter from 1 to 100 Hz and a 50 Hz Notch filter. Data were collected using the eego software with a sampling frequency of 500 Hz, while maintaining electrode impedance below 10 kΩ. The Mu rhythm was extracted at Cz in the 8–13 Hz frequency range (Wang et al., 2019, 2020).

#### Neurofeedback recording

Neurofeedback training was conducted using the BioTrace+ software (MindMedia, NeXus-10, the Netherlands), with signals acquired using a DC-coupled EEG amplifier featuring a 24-bit A/D converter to extract Mu rhythm. The amplitude of Mu rhythm was then converted into an audio-feedback tone using acoustic bass.

### Procedures

We used a stratified random control experimental design by gender to divide the population into three subgroups (TI, FSI, and SC). We followed our previous study's design as a pretest-posttest design for a single training session (Kao et al., 2014; Ros et al., 2014; Chen et al., 2022b; Wang et al., 2023). Our study incorporated three groups as a between-subject factor and employed a pre-posttest measurement as a within-subject factor. We instructed the participants to avoid consuming any food or drinks that contain alcohol or caffeine for 24 h before the day of the test. On the day of the experiment, we (a) explained the nature of the study and (b) asked the participants to sign an informed consent form. Next, we instructed them to (c) put on the Lycra electrode cap and (d) watch a 15-s putting video without any golf instruction. After that, we asked them to (e) perform a warm-up using ten balls, with the goal of putting the golf ball as accurately as possible. Then, we asked them to (f) report their attentional control and stress levels before they proceeded to (g) perform 20 putts for the pretest. After the pretest, the participants underwent (h) the EEG-NFT intervention. Following the intervention, we asked them to (i) report their attentional control and stress levels once again before they proceeded to (j) perform another 20 putts for the posttest (Figure 1). The experiment lasted ~2.5 h in total.

#### Neurofeedback training protocol

The present study followed our previous neurofeedback training protocol (Wang et al., 2023). Cortical activity was recorded from the Cz site on the EEG cap with the reference and ground electrodes attached to the left and right ear mastoids, respectively. Afterward, two training stages (i.e., pre-EEG-NFT, acquisition) were carried out. In the pre-EEG-NFT stage, we (a) asked the participants to perform ten putts for warm up, (b) averaged Mu amplitude over the ten putts, which defined as the individual training criteria (training baseline) for each participant, (c) then calculated + 20% of the baseline as a training target for the TI group and FSI group (Chen et al., 2022b; Wang et al., 2023). The instruction given to TI group and FSI group was “the computer will play a tone (auditory feedback) and display the signals on a screen (visual feedback) that is linked to your brain activity. Visual feedback is visual output from a system, such as a computer game, that allows you to interact better with the system. Auditory feedback is auditory output from a system. When you reach a prescribed level of brain activity (Mu amplitude), the tone will turn on. It represents that you are in the state that we are training for, and you need to remember the feeling that you experience when you receive the feedback”.

Importantly, two customized instructions were used in TI group and FSI group. In the TI group, the customized instruction was “Please develop your own strategies to control the brain wave”. However, in the FSI group, we asked participants to focus on their core component action (e.g., the ball path, clubface, or shoulders), that is, the action highly associated with the golf-putting task (Wang et al., 2021) and then asked them to gradually decrease attentional control of actions as increase Mu power at Central region (Wang et al., 2023). Nonetheless, random feedback was conducted for the SC group in each training trial. To guarantee the randomized feedback, we randomly played the feedback tone using random.org.

To ensure that EEG-NFT learning could be achieved, the participants were required to meet a successful training ratio of 70% (Gruzelier, 2014) in a single training trial (40 s), which was defined as the amount of time that the participant successfully entered the training threshold during the motor preparation period. If participants did not achieve the training ratio of 70%, the Mu power baseline would be increased by 10% until the training ratio of 70% was achieved.

**Figure 2:**
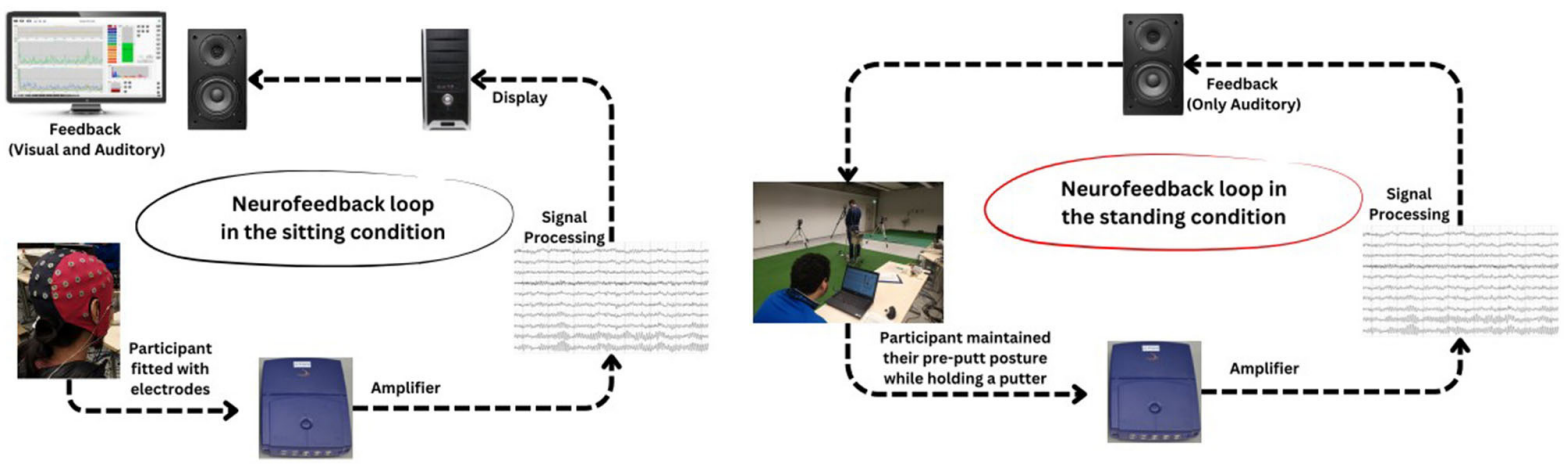
Flowcharts present neurofeedback training protocols in sitting and standing conditions.

During the acquisition stage, we utilized two distinct conditions—sitting and standing—as recommended by Kao et al. (2014) to progressively simulate putting conditions for participants in groups TI and FSI (see Figure 2). To enhance the EEG-NFT efficacy, we gradually adjusted the training threshold by +10% in the sitting condition and +20% in the standing condition. To evaluate the level of control achieved by the participants over their EEG, a successful training ratio of 80% was established. In other words, a higher ratio would indicate that the participant had better control over their EEG. During the sitting condition, the participants were instructed to sit 60 cm away from a computer monitor. To deem the training successful, the average training ratio had to be above 80% for three consecutive trials, with a minimum of six blocks of audiovisual feedback provided to the participants. Once the participants achieved the successful training criteria in the sitting condition, they were permitted to proceed to the standing condition. During the standing condition, the participants were required to maintain their pre-putt posture while holding a putter. The visual feedback was removed during the standing condition to allow the participants to engage in a pre-putt routine similar to real-life. The training protocol was identical to that of the sitting condition, and the participant had to achieve an average successful training ratio >80% for three consecutive trials of at least six blocks before they could move on to the post-task assessments.

### Data analysis

#### Behavioral data

To evaluate the performance outcomes, the researchers used a pre-posttest mean radial error (MRE) to measure putting accuracy, as described by our previous EEG-NFT study (Wang et al., 2023). MRE is defined as the average distance in millimeters that each subject's putt outcome deviated from the center of the target. A higher MRE indicates a larger average radial error, indicating lower accuracy and performance.

#### EEG data

The EEG data underwent preprocessing using both EEGLAB functions (Delorme and Makeig, 2004) and custom MATLAB scripts (MathWorks, U.S.A.). To preprocess the EEG data, the researchers performed the following steps: (1) re-referenced the data to the averaged mastoids (A1, A2); (2) applied a bandpass filter using a basic finite infinite response (FIR) filter, ranging from 1 Hz (low-pass) to 30 Hz (high-pass); (3) extracted epochs within a time window of −3,000 to 1,000 ms before the putting activity; and (4) removed channels with bad signal quality; (5) rejecting gross artifacts (amplitudes exceeding ± 100 μV) to eliminate any potential biological artifacts (e.g., muscle activation artifacts; Wang et al., 2020). As a result, a total of three trials were rejected during both the pretest and posttest stages, with the number of rejected trials varying across the groups: FSI group = three trials pretest and three trials posttest, TI group = 0 trials pretest and 0 trials posttest, and SC = 0 trials pretest and 0 trials posttest.; (6) running independent component analysis (ICA; Runica Infomax algorithm; Makeig et al., 1995) to identify and remove components caused by blinks, eye movements, and other non-neural activity; (7) interpolating channels with bad signals. The resulting clean signals were then divided into 2-s epochs spanning a time window of −2,000 to 0 ms before the putting activity. Finally, the power spectrum between 8 and 13 Hz was calculated using the Welch estimation method with a Hanning windowing function as described by Welch (1967). The pretest trial counts for FSI, TI, and SC groups were 19.70 ± 0.67, 20.00, and 20.0 trials, respectively. Posttest trial counts were 19.70 ± 0.67, 20, and 20 trials, respectively. To ensure that differences in the number of trial counts between the groups did not influence the results, a one-way analysis of variance (ANOVA) was performed. The results showed no significant differences between the groups both pretest (*p* = 0.158) and posttest (*p* = 0.158). Thus, the unequal number of trials did not affect our findings. For brevity of reporting, only the results from the key Cz electrode, and those in its immediate surroundings (i.e., C3 and C4) are presented. We selected these electrodes because they roughly overlie the frontal lobe, which consists of primary motor cortex, the premotor cortex, and the supplementary motor areas that are related to movement programming processes, all of which have been implicated in previous EEG-based golf-putting research (Babiloni et al., 2008; Cooke et al., 2014, 2015; Wang et al., 2019, 2020).

### Statistical analysis

The behavior data and EEG data was exported from the motion capture system (Vicon Motion Systems), the eego system (ANT Neuro), and the NeXus-10 system (MindMedia). We further used SPSS 26 software for statistical analysis. Separated Mixed-design ANOVA and MANOVA were performed on our measures (more details in Results section). The alpha level was set at 0.05.

## Results

### Age

A one-way ANOVA was used for the age distributions of the three groups (FSI, TI, and S). Our data demonstrated that there was no significant effect of age, *F*_(2, 27)_ = 0.068, *p* = 0.935.

### Putting performance (mean radial error)

**Figure 3:**
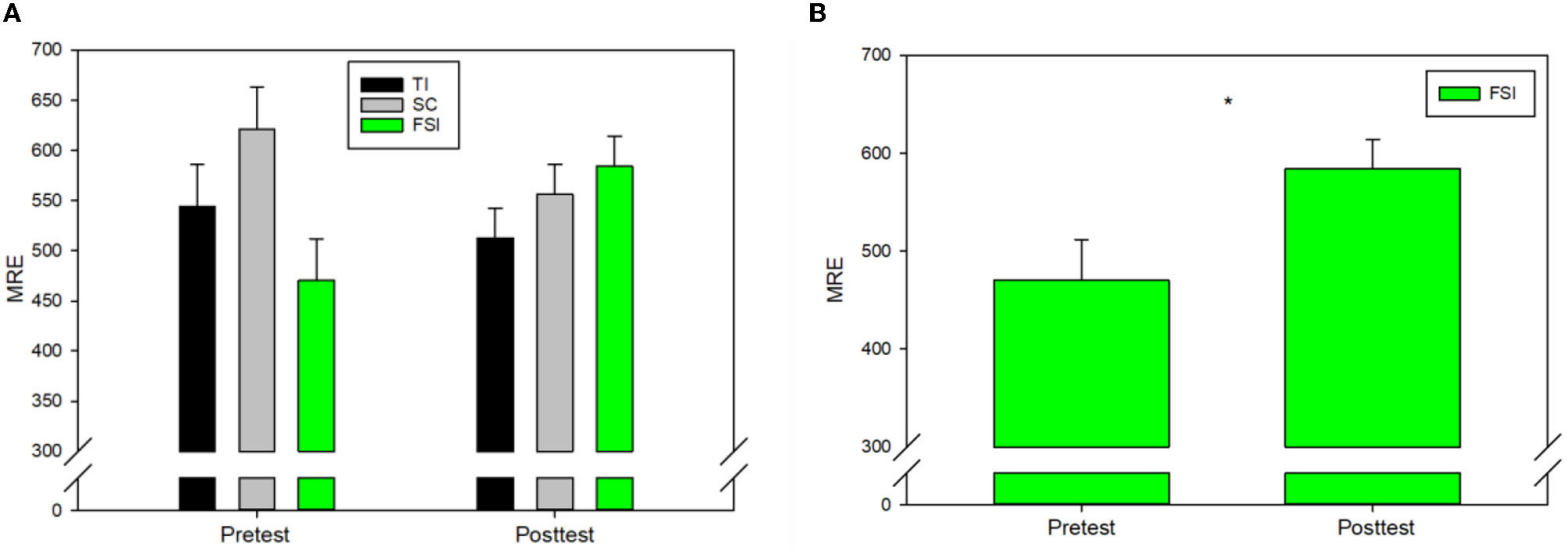
**(A)** Mean relative errors (MREs) for three groups. **(B)** FSI exhibited performance detriment after EEG-NFT. ^*^p < 0.05.

To determine the effect of EEG-NFT on golf putting performance, we ran a 3 (groups: FSI, TI, SC) × 2 (time: pretest, posttest) repeated measures ANOVA of the MRE. A repeated measures ANOVA revealed a significant interaction effect between time and group, *F*_(2, 27)_ = 8.220, *p* = 0.002, and $\eta_{P}^{2}$ = 0.378. However, no significant group effect was observed in pretest (*p* = 0.01) and posttest (*p* = 0.25, Figure 3A). Follow-up *post hoc* analysis was conducted using paired *t*-tests to indicate that that only the FSI group exhibited performance detriment after EEG-NFT (*p* = 0.013, Figure 3B).

### Subjective psychological state (attentional control level)

To examine the causal relationship between brain activity and psychological state, we ran a 3 (groups) × 2 (time) repeated measures ANOVA of the self-evaluation data (e.g., the level of attentional control). A repeated measures ANOVA revealed no significant interaction effect between time and group, *F*_(2, 27)_ = 2.313, *p* = 0.118, and $\eta_{P}^{2}$ = 0.146. Despite the non-significant result, the effect size indicates a large effect, prompting further investigation through follow-up analyses. We observed that FSI group exhibited a slight decrease in the level of action control after EEG-NFT (Pretest = 4.1 ± 1.79; Posttest = 2.9 ± 0.87) although no significant difference between pretest and posttest was observed (*p* = 0.119). Similarly, there was no significant difference between pretest and posttest in TI group (Pretest = 4.35 ± 2.21; Posttest = 3.75 ± 1.84; *p* = 0.297) and SC group (5.1 ± 2.07; Posttest = 5.8 ± 2.65; *p* = 0.332).

### Putting-State EEG

#### Brain regions

To examine the topographical specificity of 8–13 Hz at the Cz, a 3 (groups) × 2 (time) repeated measures MANOVA was carried out for the 8–13 Hz power at Fz, Cz, Pz, and Oz sites. No significant interaction was seen between group and time, *F*_(8, 48)_ = 0.570, *p* = 0.570, Wilks' lambda = 0.769, $\eta_{P}^{2}$ = 0.123, and power = 0.344.

#### Frequency bands

To examine frequency specificity, we ran a 3 (groups) × 2 (time) repeated measures MANOVA of 4–7 Hz, 8–13 Hz, and 14–20 Hz power at Cz. We confirmed that there was no significant group and time interaction, *F*_(6, 50)_ = 1.359, *p* = 0.249, Wilks' lambda = 0.739, $\eta_{P}^{2}$ = 0.140, and power = 0.482.

#### Variation in Mu activity

**Figure 4:**
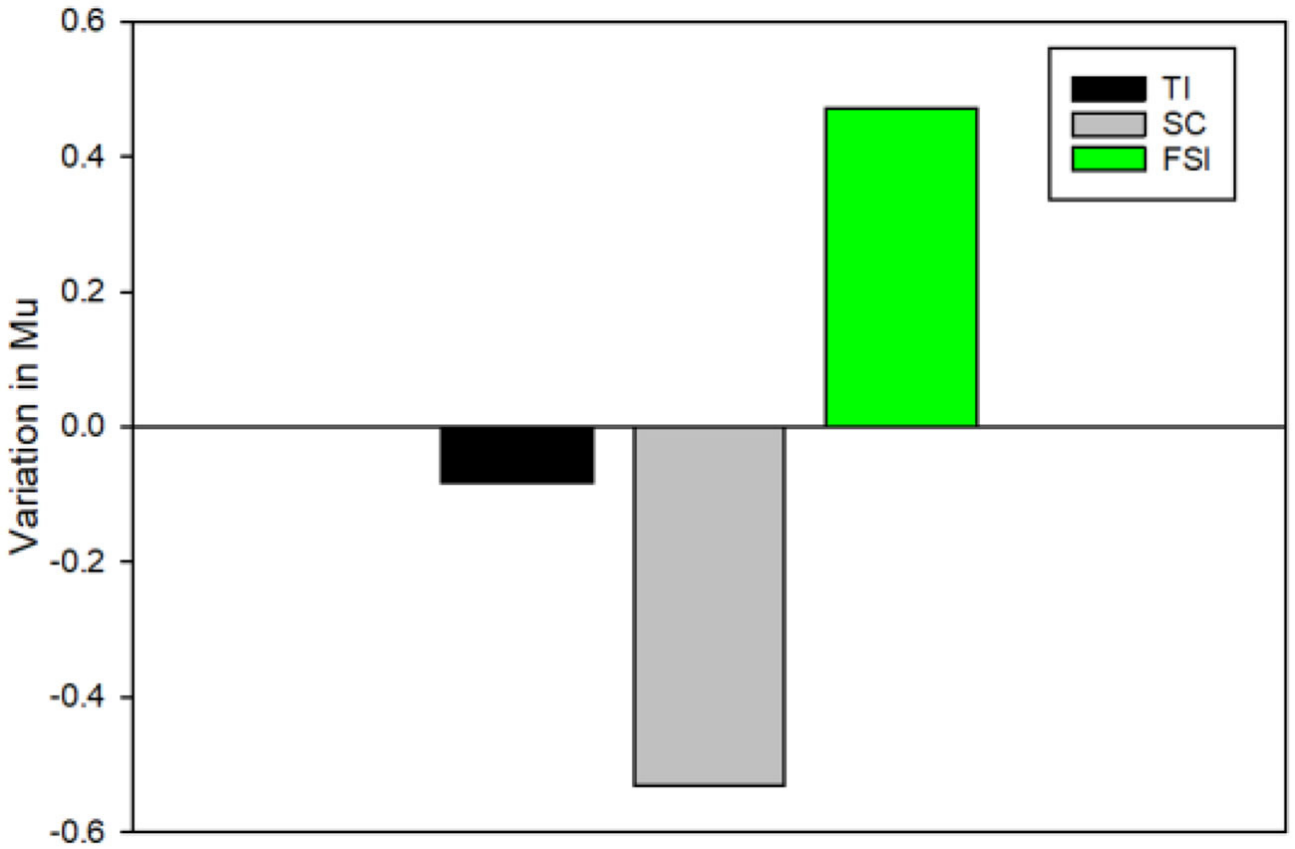
The variation in Mu activity was conducted by subtracting Mu power recorded in the posttest from Mu power recorded in the pretest. Positive value, an increase in Mu activity from the pretest to the posttest; negative value, a decrease in Mu activity from the pretest to the posttest.

To examine the variation in Mu activity, we conducted a comparison by subtracting Mu power recorded in the posttest from Mu power recorded in the pretest. This analysis allowed us to assess the tendency of Mu power alterations after EEG-NFT. We conducted a one-way ANOVA and found no significant difference in Mu activity among the groups, *F*_(2, 27)_ = 1.713, *p* = 0.199, $\eta_{P}^{2}$ = 0.113. Interestingly, we observed that only the FSI group showed a positive value (M = 0.47 ± 1.19 power), indicating an increase in Mu activity from the pretest to the posttest. In contrast, the TI group exhibited a negative value slightly (M = −0.08 ± 1.33 power), and the SC group (M = −0.53 ± 1.19 power) exhibited a negative value, suggesting a decrease in Mu activity from the pretest to the posttest (Figure 4).

### Neurofeedback training

To determine the learning effect of EEG-NFT in the FSI and TI groups, we compared 8–13 Hz power at Cz during the first and last block during the training period. A 2 (groups) × 2 (block: first and last block) repeated measures ANOVA was performed. Analyses revealed no significant interaction effect between time and group, *F*_(1, 18)_ = 0.393, *p* = 0.539, and $\eta_{P}^{2}$ = 0.021. However, analyses revealed a significant time main effect, *F*_(1, 18)_ = 14.918, *p* = 0.001, and $\eta_{P}^{2}$ = 0.453. Follow-up analyses indicated that there had significantly increased Mu power between FSI group (*p* = 0.019) and TI group (*p* = 0.029). Thus, EEG-NFT was effective in changing the targeted brain activity in novice golfers during EEG-NFT training.

### Neurofeedback training trials

To determine the effectiveness of instruction in EEG-NFT, we compared training trials in both groups. A one-way ANOVA was performed. Analyses revealed significant effect, *F*_(1, 18)_ = 5.832, *p* = 0.027, and $\eta_{P}^{2}$ = 0.245 in FSI group (Mean = 12.20 ± 0.42) and TI group (Mean = 13.1 ± 1.10). The main results indicated that the FSI approach was more effective to modulate Mu power during EEG-NFT than a TI approach.

### Correlation between changes in Mu rhythm and performance

Given that we observed the trend in the higher MRE as less accuracy and increased Mu rhythm in FSI group during golf putting, we further tested the correlation between Mu rhythm and MRE. To do so, we performed a correlational analysis between the percentage change in Mu activity and the percentage change in MRE from pretest to posttest in three groups. A Pearson's correlation analysis with a one-tailed test revealed that the percentage change in Mu activity was significantly positively correlated with the MRE (*r* = 0.319, *p* = 0.043, *N* = 30). It means that higher Mu power is correlated with higher MRE, suggesting lower putting accuracy in golf putting.

### Control analysis

To counter the confounding effects of stress, we ran a 3 (groups) × 2 (time) repeated measures ANOVA of the subjective self-reported stress levels. Self-reported subjective stress levels in pretest and posttest were compared both between and within subjects. A repeated measures ANOVA revealed no significant interaction effect between time and group (*p* = 0.212), nor any effect of time (*p* = 1.000). specifically, suggesting that stress levels did not significantly change during the course of the experiment and likely did not affect the results.

## Discussion

The objective of this study was to investigate the impact of the FSI (function-specific instruction) approach in EEG-NFT (Neurofeedback Training) and its effects on visuomotor performance (i.e., golf). The study involved manipulating Mu power (8–13 Hz at Cz) using EEG-NFT in novice golfers. Our main finding revealed that the FSI group required fewer EEG-NFT training trials compared to the TI (Traditional Intervention) group. Following EEG-NFT, we observed the FSI group demonstrated a significant decrease in performance. However, a slight increase in Mu power and a decrease in the level of the subjective sensation of action control after EEG-NFT were observed, although these changes did not reach statistical significance (*p* > 0.05) between the pretest and posttest. However, it is worth noting that these effects exhibited a large effect size ($\eta_{P}^{2}$ = 0.123–0.140), and we observed a positive correlation between Mu power and MRE, suggesting higher mu power is correlated with lower accuracy in golf putting. These results partly complement the findings of Wang et al. (2023) and highlight the potential of the FSI approach in modulating Mu activity and its implications for visuomotor performance in the context of neurofeedback training.

Regarding the effect of Mu rhythm in EEG-NFT with the FSI approach, we observed that the FSI group showed a slight increase in Mu power after a single session of EEG-NFT. Our results demonstrated a medium-large effect size ($\eta_{P}^{2}$ = 0.113) when comparing Mu activity among the three groups. However, the group effect did not reach statistical significance. The rationale of the FSI approach in EEG-NFT is that the verbal instruction should be directly related to the specific features of brain function in order to facilitate the attainment of a desired mental state within the sports context (Chen et al., 2022b). This approach aims to minimize target-irrelevant brain activity or slower timescales during the initial learning phases by explicitly guiding individuals to enter a specific mental state. This targeted instruction helps focus on and optimize the training process, enhancing the effectiveness of EEG-NFT in sports performance (Muñoz-Moldes and Cleeremans, 2020). However, our finding is inconsistent with Chen et al. (2022b) and Wu et al. (2023) who observed that skilled golfers were able to modulate their EEG activity using the FSI approach in a single training session. The possible explanation is that skill levels may be a moderator in the effectiveness of the FSI approach in EEG-NFT. For example, Chen et al. (2022b) conducted a study on EEG-NFT with skilled golfers to investigate the effects of FSI approach and TI approach. Their research revealed that a single session of EEG-NFT using frontal midline theta (4–7 Hz) training was effective in improving performance, but this effect was observed only in the FSI group, accompanied by a decrease in frontal midline theta power after training. On the other hand, the TI group did not demonstrate performance improvement and showed no significant increase in frontal midline theta power. These findings demonstrate that skilled golfers in the FSI group were able to effectively modify their neurocognitive processing and successfully translate it into improved behavioral performance after just a single session of EEG-NFT. However, the efficacy of FSI approach may not be sufficient for participants at a novice level in our current study because novices in the early stages of learning process the rules of movement (Coker et al., 2006) and engage more cognitive resources during motor preparation (Chen et al., 2022b). This inference aligns with earlier research conducted by Wang et al. (2023) who discovered that novice golfers faced challenges in augmenting Mu power using the traditional EEG-NFT approach during golf task or when engaging in novel tasks. The FSI group employed a focused approach, requiring novices to concentrate on core components of their actions (e.g., the ball path, clubface, or shoulders) that are task-relative and adjust their mental state accordingly during EEG-NFT. Despite the potential complexity of this process for novices, the results revealed a remarkable finding: participants in the FSI group achieved the training target with significantly fewer training trials compared to the TI group. This noticeable efficiency demonstrated by the FSI group highlights the potential superiority of the FSI approach in EEG-NFT, emphasizing its value for enhancing visuomotor performance. Furthermore, it is important to consider whether novices would benefit from additional training sessions such as three (Arns et al., 2008), six (Afrash et al., 2023), and eight training sessions (Cheng et al., 2015a) to consolidate their progress and ensure the retention of acquired mental skills during visuomotor tasks. This consideration will provide a more comprehensive understanding of the implications and potential optimizations for the effectiveness of the learning process in NFT training protocols (Mirifar et al., 2017; Hung and Cheng, 2018; Cheng and Hung, 2020b). Collectively, these findings provide a new perspective that the utilization of FSI approach may need to align with a certain skill level in order to exemplify its training efficacy (Wang et al., 2020; Gong et al., 2021).

Regarding the effect of performance in EEG-NFT with the FSI approach, we observed the FSI group demonstrated a significant decrease in performance. Notably, this finding aligns with previous EEG-NFT studies, showcasing the consistency of the FSI approach in modulating performance outcomes (Kao et al., 2014; Chen et al., 2022b; Wang et al., 2023). Previous NFT studies have shown that a single session EEG-NFT can alter the performance of skilled golfers (Kao et al., 2014; Chen et al., 2022b) as well as novice golfers (Wang et al., 2023). Our study extended the findings of a previous study in skilled golfers (Chen et al., 2022b) by demonstrating that EEG-NFT with the FSI approach could also have an impact on the performance of novice golfers, although we did not observe a significant group effect in Mu activity. Interestingly, we observed a correlation between higher Mu power and decrement in putting performance (higher MRE). This finding could be explained by neurophysiological evidence suggesting that Mu rhythm reflects the allocation of cognitive resources to motor programming during the execution of goal-directed actions (Pineda, 2005; Cannon et al., 2014). Specifically, higher Mu power may indicate inhibitory motor programming, reflecting a transition from conscious control to a less conscious control process as reduced sensation of attentional control on the movement before action (Pfurtscheller, 2003; Klimesch et al., 2007).

On the other hand, lower Mu power may indicate the facilitation of task-relevant motor programming as increased attentional control on the movement during motor preparation (Cooke et al., 2015). Given that golf putting is a complex visuomotor task, reduced attentional control may not be beneficial for superior performance output (Babiloni et al., 2008; Cooke et al., 2014, 2015; Wang et al., 2023). Conversely, simple visuomotor tasks like shooting may benefit from lower levels of attentional control (Haufler et al., 2000; Del Percio et al., 2009; Bertollo et al., 2016; Cheng et al., 2017). That is, performance in the complex visuomotor task may be modulated by the subjective sensation of action control. Our study found a slight decrease in the subjective sensation of action control and an increase in Mu power among FSI group participants after EEG-NFT. Although not statistically significant, the effect size was substantial (η^*P*2^ = 0.146). This suggests that novice golfers may face challenges in generating precise motor output during a complex task like golf putting due to reduced attentional control. According to the stages of learning theory, conscious control of movement is crucial during the cognitive stage, particularly for novice individuals (Schmidt et al., 2018). This aligns with previous research indicating that novices allocated more attentional resources to motor control in order to improve performance in golf (Perkins-Ceccato et al., 2003). However, an interesting question is whether there is a minimum value of the subjective sensation of action control required for superior performance during a golf putting task. To answer this question, we encourage future research to investigate the correlation between different levels of the subjective sensation of action control and performance outcomes in the context of a golf putting task. Overall, our findings suggest that manipulating Mu rhythm through EEG-NFT with FSI approach can lead to performance alternation in complex visuomotor tasks, such as golf putting, although no significant group effect in Mu activity was observed. This finding may support the notion that a small change in cortical activity can translate into an alteration in performance (Vernon, 2005; Cheng et al., 2015a; Aloufi et al., 2021). However, higher Mu power in the brain may impede the execution of complex visuomotor task, while it may be benefiting the execution of simple visuomotor task, such as shooting. Further research is needed to confirm how different motor representations (simple vs. complex visuomotor skills) can modulate Mu activity during motor preparatory processes (Berka et al., 2010) and whether increased Mu activity may benefit the execution of simple visuomotor tasks.

The potential impact of stress (a confounding factor) on putting performance and Mu rhythm was investigated in our study, and it was found that stress levels did not have a significant effect on participants. Control analysis revealed no significant changes in stress levels among the three groups or between the pretest and posttest, indicating that stress remained stable throughout the experiment and likely did not influence the outcomes. Previous studies have highlighted the importance of studying the relationship between mental stress, brain dynamics, and motor performance under challenging stressors to assess the potential induction of increased connectivity (Lo et al., 2019). Hatfield et al. (2013) observed desynchrony of high-alpha power (10–12 Hz) during a competitive pistol shooting match, suggesting heightened attentional processing and reduced neural efficiency under stress conditions. The observed implication is that mental stress has the potential to negatively affect cognitive-motor performance by interfering with the coordination of sensorimotor processes and increasing the effort required for task execution (Lo et al., 2019). This finding aligns with the psychomotor efficiency hypothesis proposed by Hatfield (2018) and is further supported by studies utilizing the multi-action plan (MAP) model (Bertollo et al., 2016). Extensive research has highlighted the negative impact of excessive cognitive processing on skilled performance, leading to issues like degraded motor performance, altered motor unit activity, and reduced accuracy (Lo et al., 2019; Cheng et al., 2022). To address potential confounding factors, our study diligently controlled stress levels within and between the three groups during pretest and posttest assessments. Consequently, the observed disparities in putting performance and Mu rhythm activity between the FSI and TI groups were less susceptible to stress-related influences and more plausibly attributed to the distinct effects of the FSI approach on visuomotor performance.

Several limitations and recommendations can be discussed based on the findings of our current study. Firstly, we acknowledge the lack of statistical significance in our Mu activity during the golf putting task and subjective psychological state findings, despite observing trends and large effect sizes. This highlights the need for larger sample sizes or modifications to the experimental design to strengthen the statistical power of future studies. To address this limitation and improve the generalizability of our results, it is recommended that future studies include larger sample sizes, considering individual differences and potential subgroups within the participant pool. Secondly, our findings suggest that the effectiveness of the FSI approach in EEG-NFT may be influenced by the skill level of participants. Novice golfers in our study may not have fully benefited from the FSI approach due to their early stage of learning and limited ability to modify their neurocognitive processing. Therefore, future research should explore the interaction between skill level and the efficacy of the FSI approach. This investigation will provide a deeper understanding of how skill level moderates the effectiveness of FSI approach in EEG-NFT. Lastly, the generalizability of our findings is limited as our study specifically focused on the impact of FSI approach in EEG-NFT on visuomotor performance in novice golfers. To further neurofeedback research in sports, future studies must rigorously investigate the most effective EEG markers, especially in the context of golf putting. Recent investigations, such as those conducted by Afrash et al. (2023) and Pourbehbahani et al. (2023), have underscored the benefits of enhanced sensorimotor rhythm (SMR) and suppressed alpha neurofeedback in facilitating long-term motor learning among novice golfers. Cheng et al. (2015a) extended this research by correlating higher sensorimotor power at the Cz point with enhanced performance in skilled golfers. This body of work underlines the need for comprehensive research to pinpoint and validate the most efficacious EEG markers. Focused research is imperative for devising accurate and impactful neurofeedback training methods in sports, particularly to improve complex visuomotor skills, such as golf. Additionally, understanding how the FSI approach can be adapted and refined for various sports, ranging from simple visuomotor tasks like shooting to complex ones like golf putting, archery, and penalty kick (Chang and Hung, 2020), and for different skill levels from novices to experts, is crucial. Furthermore, adopting long-term training protocols is crucial to explore the lasting effects of FSI approach in EEG-NFT and assess the retention of acquired mental skills. Future studies could incorporate extended training interventions, such as 8-session training programs (Cheng et al., 2015a; Christie et al., 2020), to evaluate the durability and transferability of these skills. This will provide valuable insights into the long-term benefits of FSI approach in EEG-NFT. Collectively, addressing the limitations of our study and pursuing these recommendations will contribute to a more comprehensive understanding of the impact of the FSI approach in EEG-NFT, its efficacy across different skill levels, and its potential applications in enhancing visuomotor performance.

The results of our study offer meaningful implications for real-life application in sports coaching and practice. Coaches and practitioners can utilize these findings to design more effective and targeted training programs. Our study suggests that the effectiveness of the FSI approach in EEG-NFT may vary depending on the skill level of participants. Novice golfers in our study did not fully change their EEG activity and mental state during visuomotor task from the FSI approach, possibly due to their early stage of learning and limited ability to modify their neurocognitive processing. Coaches and practitioners should take into account the skill level of athletes when implementing the FSI approach in EEG-NFT, as it may be more effective for skilled individuals who can effectively modify their neurocognitive processing and translate it into improved performance. Secondly, our study highlighted the different effects of attentional control on performance in complex visuomotor tasks like golf putting compared to simple visuomotor tasks like shooting. Reduced attentional control may be detrimental to performance in complex tasks but beneficial in simple tasks. Coaches and practitioners should be aware of the subjective sensation of action control required for different tasks and tailor training approaches accordingly. This understanding will assist in optimizing performance outcomes and facilitating skill development. Lastly, for long-term training protocols, our findings indicated that participants in the FSI group required fewer training trials to reach the training target compared to the traditional intervention (TI) group. To enhance the effectiveness and retention of acquired mental skills during visuomotor tasks, coaches and practitioners may consider implementing extended training protocols, such as 8-session interventions (Cheng et al., 2015a; Hung and Cheng, 2018; Cheng and Hung, 2020a). This will allow novice athletes to consolidate their progress and ensure the long-term benefits of FSI in EEG-NFT (Mirifar et al., 2017; Xiang et al., 2018; Onagawa et al., 2023). In summary, our study emphasizes the importance of considering skill level, understanding the complexity of visuomotor tasks and implementing long-term training protocols when utilizing the FSI approach in EEG-NFT. These insights can guide coaches and practitioners in designing scientific training programs that effectively enhance visuomotor performance.

This study aimed to investigate the impact of implementing the function-specific instruction (FSI) approach in EEG neurofeedback training (NFT) on visuomotor performance, specifically in the context of golf. The study highlights the potential of the FSI approach in EEG-NFT for influencing brain activity and visuomotor performance. However, the effectiveness of the FSI approach in EEG-NFT may vary depending on participants' skill level. Novices may need additional training sessions in EEG-NFT with FSI approach to consolidate progress and retain acquired mental skills. In complex visuomotor tasks, attentional control may play a crucial role, as higher Mu power correlates with lower performance. To conclude, these findings provide valuable insights for optimizing EEG-NFT protocols and enhancing visuomotor performance. Recommendations for future studies include exploring skill-level specificity, implementing long-term training interventions, and studying diverse populations and motor tasks.

## Data availability statement

The original contributions presented in the study are included in the article/supplementary material, further inquiries can be directed to the corresponding author.

## Ethics statement

The studies involving humans were approved by Ethics Committee of Bielefeld University. The studies were conducted in accordance with the local legislation and institutional requirements. The participants provided their written informed consent to participate in this study.

## Author contributions

K-PW: Conceptualization, Funding acquisition, Investigation, Methodology, Software, Supervision, Writing – original draft, Writing – review & editing. M-YC: Conceptualization, Methodology, Validation, Writing – original draft, Writing – review & editing. HE: Data curation, Investigation, Methodology, Validation, Writing – review & editing. TS: Resources, Validation, Writing – review & editing.
